# Discovery of N-[2-(4-methylquinolin-2-yl)phenyl]acetamidine as a new potent nitric oxide synthase inhibitor against glioma progression

**DOI:** 10.1007/s11030-025-11309-0

**Published:** 2025-08-05

**Authors:** Marialucia Gallorini, Rosa Amoroso, Amelia Cataldi, Cristina Maccallini

**Affiliations:** Department of Pharmacy, University “G. d’Annunzio” of Chieti-Pescara, Chieti, Italy

**Keywords:** Anticancer, Drug discovery, Dual inhibitors, Glioma, Hybridization, Nitric oxide synthase, Synthesis

## Abstract

Gliomas are aggressive brain tumors with limited treatment options, often leading to poor patient outcomes despite surgery, radiation, and chemotherapy. Current therapies, such as temozolomide and radiation, provide only temporary control, as gliomas frequently develop resistance. Therefore, there is an urgent need for new therapeutics to improve survival and quality of life for patients. In the present study, we explore the hypothesis that the dual inhibition of both the neuronal and inducible nitric oxide synthases could represent a promising therapeutic approach, being these two enzymes often dysregulated in gliomas. To this end, the new quinoline-based compound **3** was synthetized by a simple, innovative and solvent-free procedure. The molecule was a potent dual inhibitor and demonstrated significant antitumor activity against glioma, both as a monotherapy and in combination with temozolomide.

**Graphical abstract:**

The new quinoline-based compound **3** was synthetized by a simple, innovative and solvent-free procedure. The molecule was a potent dual nitric oxide synthase inhibitor and demonstrated significant antitumor activity against glioma, both as a monotherapy and in combination with temozolomide.

**Figure:**
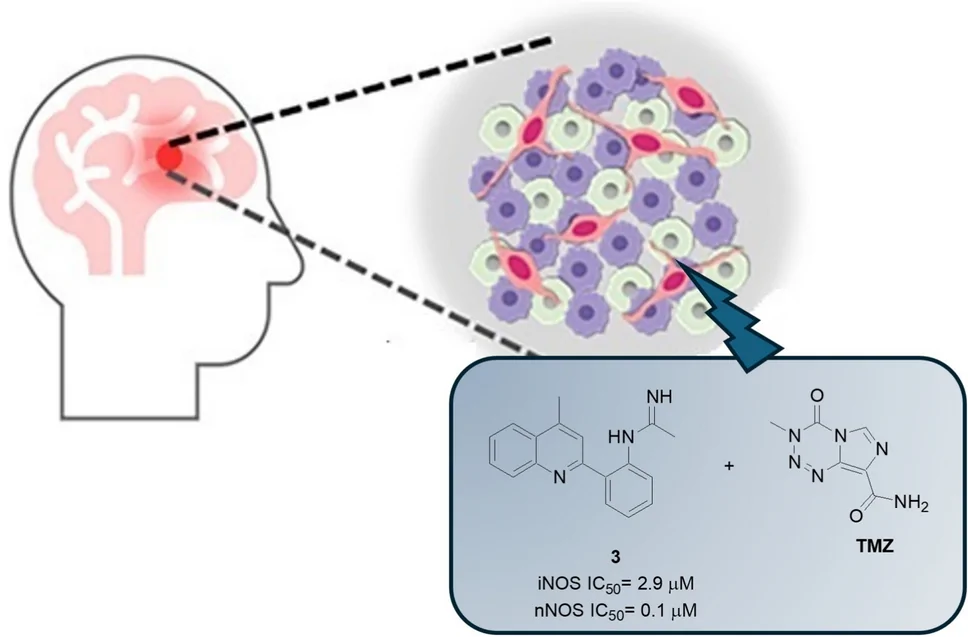

**Supplementary Information:**

The online version contains supplementary material available at https://doi.org/10.1007/s11030-025-11309-0.

## Introduction

Gliomas are a group of primary brain tumors that originate from glial cells, which provide support and protection for neurons in the central nervous system (CNS). Gliomas represent the most common type of primary brain tumor, accounting for approximately 30% of all brain tumors and around 80% of malignant brain tumors [1]. There are different types of gliomas, such as astrocytoma, oligodendrogliomas, and ependymomas, and each type can be further categorized by grade, reflecting the tumor's aggressiveness: low-grade (Grade I and II, according to the World Health Organization (WHO)) tumors tend to grow slowly and may not be immediately life-threatening, while high-grade (WHO Grade III and IV) tumors, such as glioblastoma multiforme, are highly malignant and associated with poor prognosis due to their rapid growth and invasive nature [2]. Chronic inflammation is a significant contributor to glioma growth, since it facilitates tumor promotion and neo-vascularization, which are critical for its survival and expansion [3, 4]. Glioma cells can modulate immune responses, leading to an immunosuppressive microenvironment that facilitates tumor growth.

The inducible isoform of nitric oxide synthase (iNOS) is often overexpressed in gliomas. This enzyme is deputed to the biosynthesis of nitric oxide (NO) and it is typically induced in response to inflammatory stimuli such as cytokines and microbial products to produce large amounts of NO. This is a free radical molecule endowed with multiple physiopathological functions. In the innate immune response, it can help to eliminate pathogens, but excessive NO production can lead to tissue damage and contribute to inflammatory diseases. This dualism is observed also in gliomas, where NO can induce apoptosis at low concentrations, but may also support tumor cell survival and resistance to treatments like chemotherapy and radiotherapy under certain inflammatory conditions [5]. The implications of iNOS in cancer biology are quite complex, and they do not rely only on NO concentration, but also on tumoral microenvironment and on cellular context, as reviewed elsewhere [6, 7]. Consequently, the inhibition of iNOS has garnered significant attention as a potential therapeutic strategy for gliomas. Previous in vitro studies have demonstrated that compounds such as N_G_^-monomethyl–L–arginine^ (L-NMMA), CM544, FAB1020, and 1400W can reduce NO levels, inhibit glioma cells proliferation, and enhance the effectiveness of standard treatments like temozolomide (TMZ) [8].

Besides iNOS, there are two constitutive isoforms, i.e., the neuronal NOS (nNOS) and the endothelial NOS (eNOS). The eNOS-derived NO is a key regulator of vascular tone, promoting relaxation of smooth muscle cells in blood vessels, thereby facilitating increased blood flow and reducing blood pressure. The nNOS is involved in neurotransmission within the central nervous system, influencing synaptic plasticity and neuronal communication [9]. It has been reported that the expression of nNOS is significantly increased in gliomas, correlating with both histological grade and the proliferative potential of the tumors [10]. Moreover, a recent study has reported that the treatment of a U87-MG glioma cell line with the iNOS inhibitor N6-(1-iminoethyl)-L-lysine (L-NIL), or 7-Nitroindazole (7-NI), an nNOS inhibitor, led to a reduction of clonogenic proliferation and nitrosative stress [11].

**Fig. 1:**
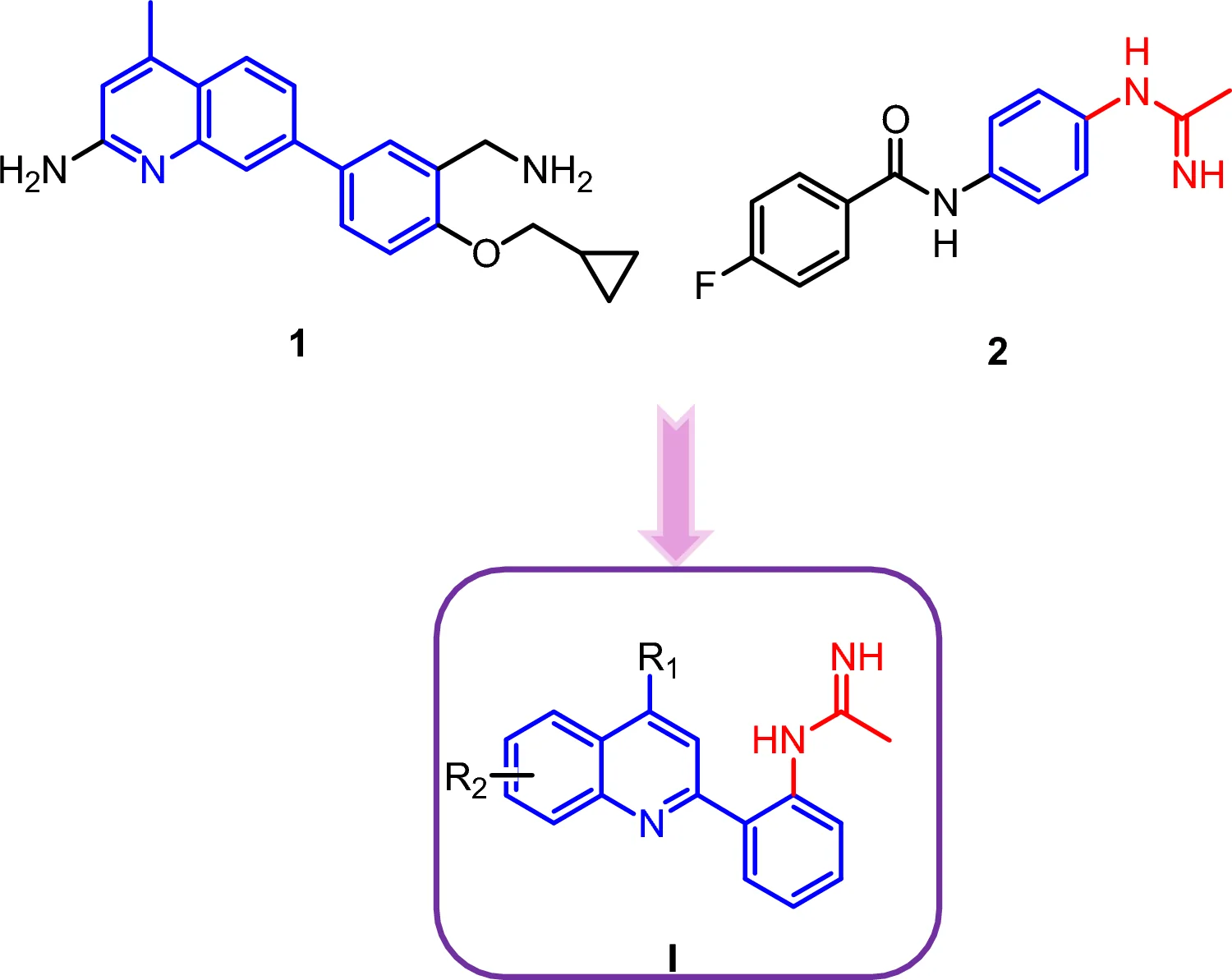
Design of structure I by means of the hybridization of the nNOS inhibitor 1 with the iNOS inhibitor 2

Based on the involvement of both iNOS and nNOS in glioma biology, in the present work we have hypothesized that a dual inhibitor targeting both enzymes could serve as a more effective therapeutic strategy than agents inhibiting either iNOS or nNOS alone. This strategy indeed, previously allowed to identify new compounds efficacious in models of neuropathic pain and inflammation. [12, 13]. To target both the nNOS/iNOS isoforms while preserving the physiological activity of eNOS, we hybridized the quinoline scaffold, previously shown to be effective in nNOS inhibition [14], with the acetamidine moiety, a key pharmacophore found in several potent iNOS inhibitors [15–17] (Fig. 1).

Here we describe the synthesis of a molecule related to the general structure I (compound **3**) by a simple, innovative and solvent-free procedure and its biological evaluation as a promising anti-glioma agent.

## Results and discussion

### Chemistry

**Scheme 1.**
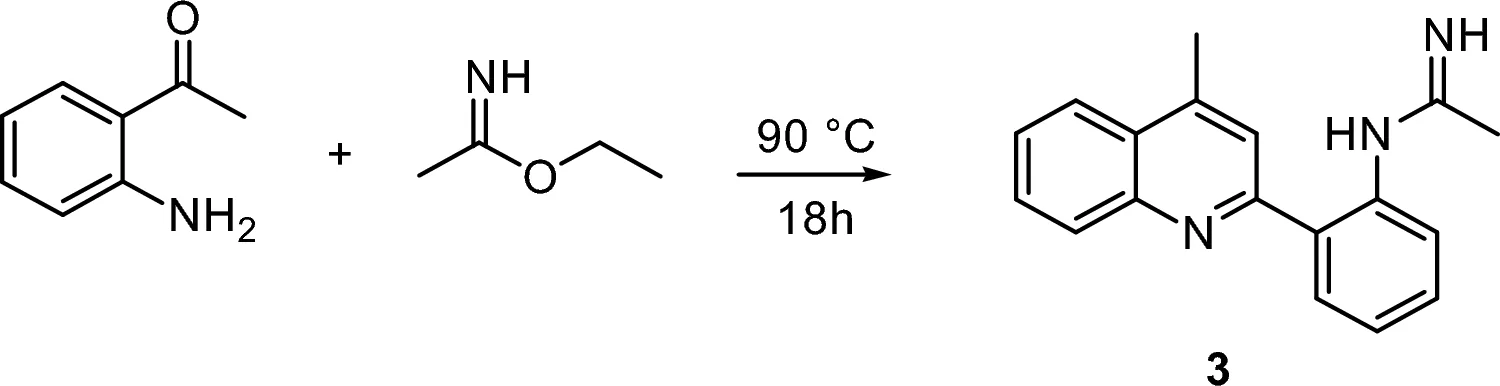
Synthesis of 3. Conditions: solvent-free; 90 °C, 18 h, 56% yield

To obtain compound **3**, a multicomponent solvent-free synthesis approach was used (Scheme 1), in which 2 equivalents of the 2-aminoacetophenone were reacted with 1 equivalent of ethyl acetimidate hydrochloride. The reaction proceeded in both ethanol and solvent-free conditions, although the last gave the best yield (see Table S1).

**Scheme 2.**
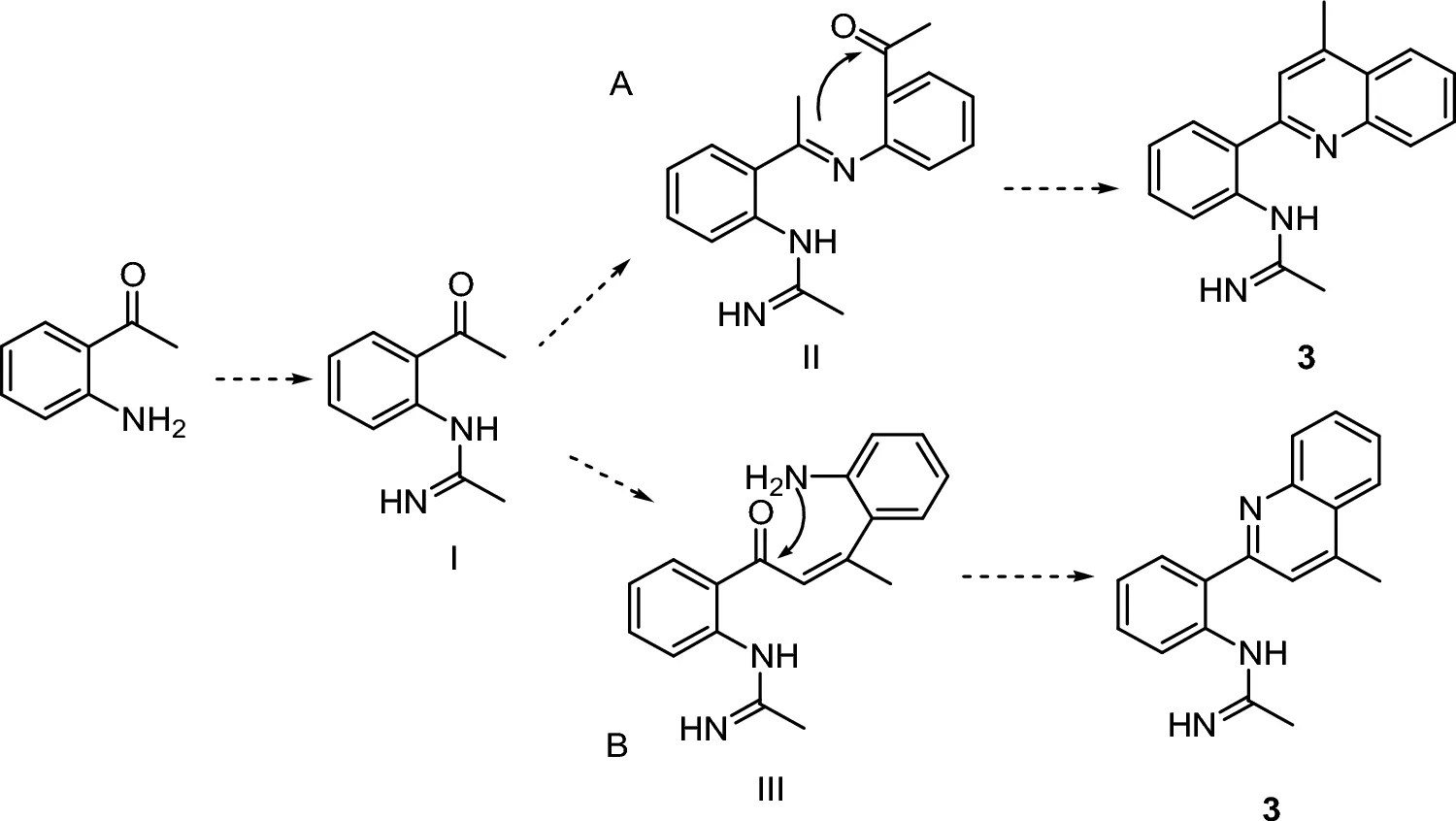
Hypothesis for the Friedländer-type reaction mechanism involved in the formation of **3**

In particular, we hypothesized that the ethyl acetimidate hydrochloride catalyzed a Friedländer-type reaction between the 2-aminoacetophenone molecules giving **3**, according to Scheme 2. The initial reaction between the ethyl acetimidate with 2-aminoacetophenone gives an intermediate I that can react with a second molecule of 2-aminoacetophenone following mechanism A or B. In the first case, an imine intermediate II is formed. followed by an intramolecular aldol condensation. Mechanism B passes through a crossed aldol condensation furnishing amino chalcone III, followed by formation of an imine to construct the target quinoline **3**.

### Biology

#### Nitric oxide synthase activity

Compound **3** was evaluated as NOS inhibitor by using the L-citrulline assay with fluorimetric detection [17]. All three isoforms were assayed, in order to evaluate isoform selectivity toward the eNOS. Indeed, this constitutive enzyme participates in vascular homeostasis, and its inhibition must be avoided. As from the results shown in Table 1, nNOS appeared the most inhibited isoform, with an IC_50_ value of 0.1 ± 0.01 µM, resulting in a complete enzyme inhibition at 1 µM. At the same time, also iNOS was inhibited, although in a less extent (IC_50=_ 2.9 ± 0.09 mM), while the eNOS activity was preserved. Comparing these results to those obtained from the parent iNOS inhibitor **2**, it can be inferred that the quite potent nNOS inhibition can be related to the presence of the quinoline pharmacophore, confirming the rationale beyond the drug design.

**Table 1:** NOS inhibition and selectivity by target compound **3**. Compound **2** is the reference compound

Compound	Inhibition (%)^a^			IC_50_ (µM)^a^			Selectivity^d^	
	iNOS^b^	nNOS^b^	eNOS^c^	iNOS	nNOS	eNOS	eNOS/nNOS	eNOS/iNOS
**3**	82 ± 3	100	n.a	2.9 ± 0.09	0.1 ± 0.01	> 100	> 250	> 34
**2**	100	n.a	n.a	0.01 ± 0.01	6.2 ± 0.08	> 100	> 16	> 1000

^a^Values given are mean ± SD of three experiments

^b^evaluated in the presence of 1 µM concentration of each compound

^c^evaluated in the presence of 10 µM concentration of each compound

^d^Expressed as the IC_50_ ratio of the relevant NOS isoforms

*n.a*. not active

### Drug-likeness and pharmacokinetic profile

As absorption, distribution, metabolism and excretion (ADME) profile is an important basis for desirable therapeutic outcome of drugs, pharmacokinetic properties of **3** were calculated by SwissADME online software [18]. As listed in Table 2, compound **3** follows the drug-likeness Lepinski’s, Veber’s and Muegge’s rules and shows promising pharmacokinetics, e.g., high gastrointestinal absorption and Blood Brain Barrier (BBB) penetration. Moreover, microsomal stability was evaluated according to a literature method [19]. Based on the obtained data, the compound shows a reasonably metabolic stability, with a 73% remaining of the parent molecule after incubation in liver microsomes at 37 °C for 60 min. Encouraged by the promising preliminary data, further preclinical investigation of **3** in a glioma model were performed.

**Table 2:** Evaluation of compound **3**’s pharmacokinetic parameters

Parameter	Value
Num. rotatable bonds^a^	3
Num. H-bond acceptors^a^	2
Num. H-bond donors^a^	2
TPSA^a^	48.77 Å^2^
cLogP^a^	3.66
GI absorption^a^	High
BBB permeant^a^	Yes
P-gp substrate^a^	No
Microsomal stability^b^	73%
Lepinski’s, Veber’s and Muegges’s rule violation	NO (0 violation)
PAINS^c^	0 alert

^a^predicted

^b^evaluated. Percent (average, n ≥ 3) remaining of the parent molecule after incubation at 37 °C for 60 min

^c^Pan-assay interference structures; predicted

### Assessment of cell viability and cytotoxicity in normal rat astrocytes (CTX) and C6 rat glioma cells

**Fig. 2:**
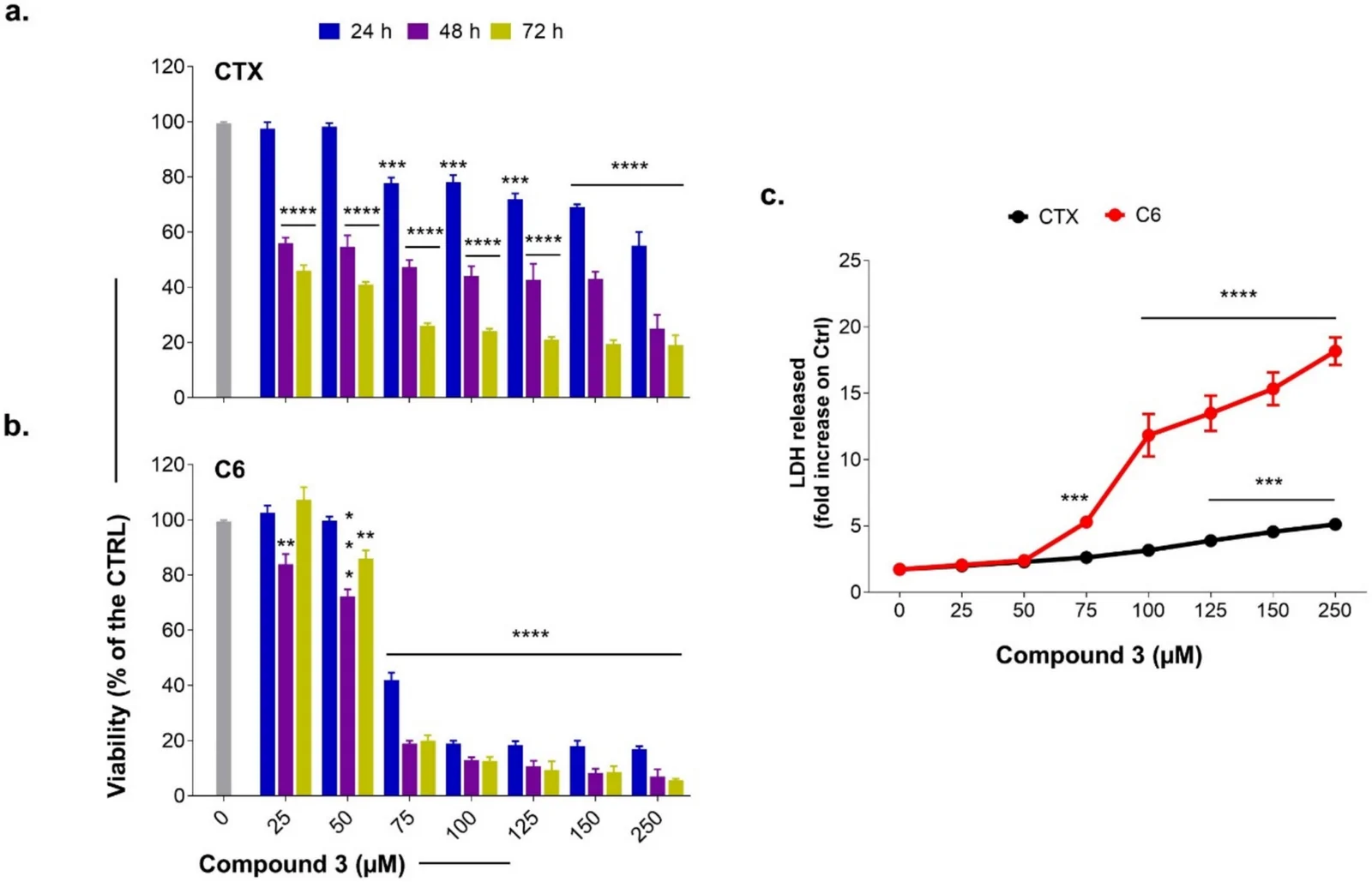
Cell viability assessment in the presence of increasing concentrations of compound 3. Bar graphs represent percentages of cell viability after the exposure of CTX (**a**) and C6 (**b**) cell lines to compound 3 for 24, 48 and 72 h. The untreated control (CTRL = 0 µM) is set as the 100%. **c** Trend lines show the LDH released from CTX and C6 cells after 24 h in the presence of compound 3. Data are presented as the fold increase on the untreated control (CTRL = 0 µM). **p < 0.01, ***p < 0.001 and ****p < 0.0001 between test samples and the untreated control

To assess the biological effect of **3**, increasing concentrations of the compound were administered on two rat cell lines, namely CTX and C6. CTX TNA2 is an astrocyte, type I cell line that was isolated from the cortex of 1-day-old rats, and it is widely used as a cell model for neurological studies [20]. The Ca^2+^-dependent NO-synthesizing enzymes eNOS and nNOS are constitutively expressed in astrocytes, whereas the expression of iNOS is typically assumed to be induced by cytokines and other agents during the immune response, and its activity does not depend on an increment in [Ca^2+^]_i_ [21]. Activated astrocytes can contribute to the development of gliomas, where the iNOS has been demonstrated to be overexpressed [8]. Against this background, the rat CTX TNA2 and the C6 rat glioma cell lines were used to assess the biological activity of compound **3** in terms of cell viability and cytotoxicity occurrence (Fig. 2). Increasing concentrations of **3** were administered on both the cell lines for 24, 48 and 72 h. After the exposure to **3**, viability percentages related to CTX decrease time-dependently and dose-dependently (Fig. 2a). After 24 h of exposure, this trend starts at 75 µM, whereas after 48 and 72 h, cell viability is lower than 60% already at 25 µM. When C6 rat glioma cells are exposed to **3** (Fig. 2b), percentages of cell viability dramatically fall to values lower than 40% starting from 75 µM at 24 h, and the significant decrease is even amplified after 48 and 72 h (percentages lower than 20%). By comparing trends of cell viability in the two cell lines, it can be highlighted that the decrease of percentages caused by **3** in normal astrocytes is slower than the one occurring in the glioma cell line. Likewise, the release of lactate dehydrogenase (LDH) and thus cytotoxicity occurrence is dramatically enhanced in C6 cells if compared to the one occurring in CTX at the same concentrations and time of exposure (Fig. 2c). These results may reflect data of NOS inhibition (Table 1) which highlight that **3** is active on both the nNOS and the iNOS, suggesting an additive effect of the compound on glioma cells.

**Fig. 3:**
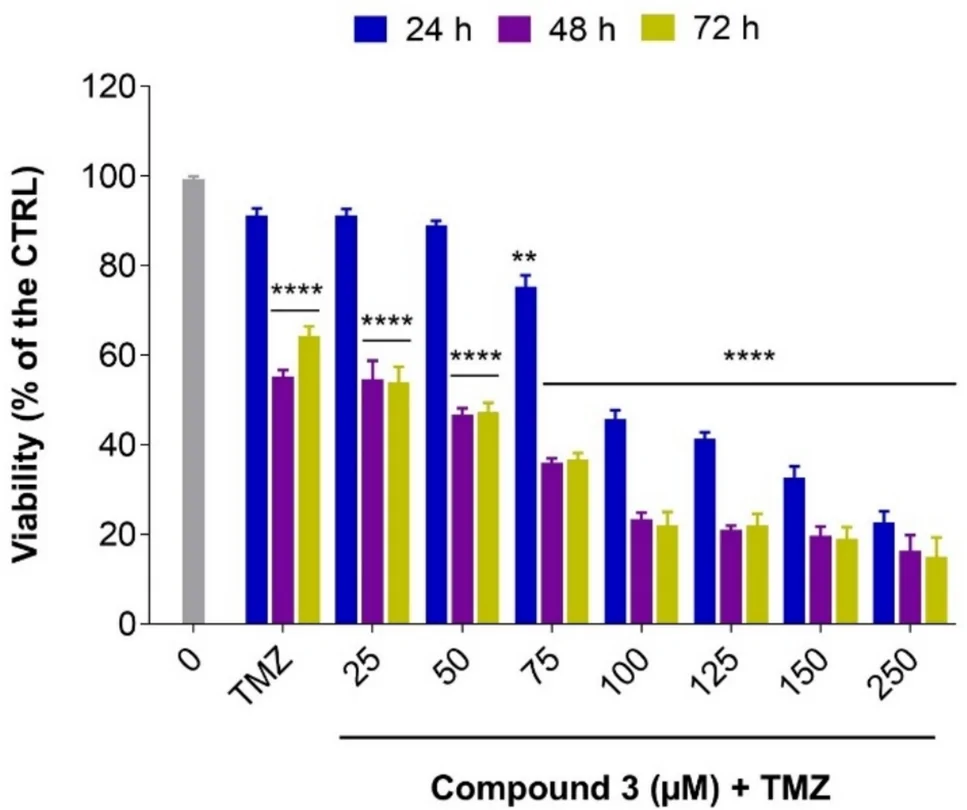
Cell viability assessment in the presence of increasing concentrations of compound 3 and Temozolomide (TMZ) after 24, 48 and 72 h. TMZ was administered at 500 µM. Bar graphs represent percentages of cell viability after the exposure of C6 cells. The untreated control (CTRL = 0 µM) is set as the 100%. **p < 0.01 and ****p < 0.0001 between test samples and the untreated control

To investigate whether a synergistic effect might be obtained between TMZ and **3**, both the compounds were administered only on C6 rat glioma cells (Fig. 3). TMZ was administered at 500 µM, as already reported [22]. Unexpectedly, percentages of cell viability in the presence of TMZ + **3** are higher than the ones in the presence of **3** alone. It has been widely demonstrated that glioma cells activated several mechanisms involved in TMZ-resistance, contributing to unbeneficial clinical outcomes [23]. Some works report a function of neuronal nitric oxide synthase enzyme in temozolomide-induced damage of astrocytic tumor cells [24], it might be plausible to speculate that, by acting also on the inducible isoform, compound **3** is more efficient if used alone in vitro.

## Conclusions

In conclusion, by the hybridization of different pharmacophores, a new dual nNOS/iNOS inhibitor was obtained. Compound **3** is highly accessible by means of a facile and eco-friendly synthesis and demonstrated significant antitumor activity against glioma, both as a monotherapy and in combination with TMZ. Compared to the selective iNOS inhibitors already known for their anti-glioma activity [8, 23], **3** offers a potential therapeutic advantage due to its dualism, making it effective also against gliomas with high nNOS expression. Moreover, its antiproliferative and cytotoxic effects, along with selectivity for tumor cells and a potential to improve TMZ efficacy, suggest that **3** could be considered in future therapeutic research. On the other side, to progress this scaffold toward therapeutic application, some issues remain to be addressed in future studies, such as the deeper understanding of the key molecular features driving selectivity and potency of **3**, as well as its efficacy and safety in further glioma models.

## Methods

### Chemistry

#### General methods and materials

Chromatography was performed on silica gel 60 (Merck) and TLC on silica gel 60, F254. Melting points were determined on a Buchi apparatus and given uncorrected. NMR spectra were run on a Varian instrument, operating at 300 (1H) or 75 (13C) MHz; chemical shifts (*δ*) are reported in ppm. HPLC was a Waters (Milford, MA, USA) system composed of a P600 model pump, a 2996 photodiode array detector, and a 7725i model sample injector (Rheodyne, Cotati, CA, USA). The analyses were run on an Xterra MS C8 column (250 × 4.6 mm id, 5 mm particle size) (Waters), equipped with an Xterra MS C8 guard column (Waters). A column thermostat oven module Igloo-Cil (Cil Cluzeau Info Labo, France) was used. Elemental analyses were carried out by a Eurovector Euro EA 3000 model analyzer. Analyses indicated by the symbols of the elements were within ± 0.4% of the theoretical values.

#### Synthesis of N-[2-(4-methylquinolin-2-yl)phenyl]ethanamide hydrochloride

A mixture of 2'-aminoacetophenone (0.016 mol, 1.945 mL) and ethyl acetimidate hydrochloride (0.008 mol, 1 g) was placed in a reaction flask and allowed to react under magnetic stirring, at 90 °C, for 18 h. After this time, the reaction was partitioned between H_2_O (10 mL) and ethyl acetate (10 mL). The separated aqueous layer was extracted with ethyl acetate (2 × 10 mL) and the combined organic fractions were dried over anhydrous Na_2_SO_4_ and then brought to dryness under reduced pressure. The obtained residue was purified on a silica gel column (mobile phase: dichloromethane/methanol 9:1), obtaining a yellowish solid. 56% yield. M.P. 117–118 °C. ^1^H NMR (CD_3_OD) δ: 2.19 (s, 3H); 2.81 (s, 3H); 7.28 (t, *J* = 15.3 Hz, 1H); 7.44 (t, *J* = 15.3 Hz, 1H); 7.65 (t, *J* = 15.3 Hz, 1H); 7.8 (m, 2H); 7.91 (d, *J* = 7.8 Hz, 1H); 8.09 (d, *J* = 8.1 Hz,1H); 8.15(d, *J* = 8.7 Hz,) 8.32 (d, *J* = 8.4 Hz, 1H); 12.9 (s, 1H). ^13^C NMR (DMSO) δ:18.9, 25.2, 121.6, 121.7, 123.7, 124.6, 125.5, 126.7, 127.3, 129.1, 130.5, 130.6, 138.5, 145.8, 146.7, 157.4, 168.5, 171.8. Anal calcd for C_18_H_17_N_3_: C, 78.52; H, 6.22; N, 15.26. Found C, 78.43; H, 6.28; N, 15.37.

### Biology

#### NOS inhibition assay

Recombinant human iNOS was purchased from Enzo Life Sciences, Inc. (New York, USA). Recombinant bovine eNOS was purchased from Cayman Chemical (Ann Arbor, USA). To measure iNOS activity, 10 μL of enzyme stock solution were added to 80 μL of 2-[4-(2-hydroxyethyl)piperazin-1-yl]ethanesulfonic acid (HEPES) buffer pH = 7.4, 100 mM, containing 0.1 mM CaCl_2_, 1 mM D,L-dithiothreitol (DTT), 0.5 mg/mL BSA, 10 μM flavin mononucleotide (FMN), 10 μM flavin adenine dinucleotide (FAD), 30 μM tetrahydrobiopterin (BH4), 10 μg/mL calmodulin (CaM), 10 μM L-Arg. For the eNOS activity evaluation, 25 μL of the enzyme stock solution were added to 65 μL of HEPES buffer containing 2 mM CaCl_2_ and the same cofactors cocktail used for the iNOS assay. Then, 10 μL of the test compound solution or 1400W (0.1–100 μM) were added to the enzyme assay solution, followed by pre-incubation of 15 min at 37 °C. Each reaction was initiated by the addition of 10 μL of NADPH 7.5 mM, carried out at 37 °C for 20 min, and stopped by adding 500 μL of ice-cold CH_3_CN. The mixture was brought to dryness under vacuum and eventually stored at − 20 °C, before the HPLC analysis.

#### HPLC analysis of the NOS assays

The analysis was performed according to a previously reported method [17], with minor modifications. The o-phthalaldehyde-N-acetylcysteine (OPA/NAC) reagent for fluorescence derivatization of the NOS reaction mixtures was prepared with the molar ratios of 1:3, reacting for 90 min 5 mL of methanolic OPA solution and 20 mL of 0.2 M borate buffer containing 0.1 g of NAC to final pH 9.3 ± 0.05. The OPA/NAC solution was stored at 4 °C and saved for no longer than seven days. 600 μL of HPLC grade water was added to the residue of the enzymatic assay and centrifuged at 6000 rpm for 20 min. The fluorescence reaction is realized stirring 190 μL of supernatant and 60 μL of OPA/NAC solution for 5 min. The HPLC column was eluted at a flow rate of 0.7 mL/min with linear gradients of buffers A (5% CH_3_CN in 15 mM sodium borate with 0.1% v/v TFA, pH 9.4) and B (50% CH_3_CN in 8 mM sodium borate with 0.1% v/v TFA, pH 9.4). The solvent gradient was 0–20% B at 0–10 min, B to 25% at 10–15 min, then to 40% at 15–20 min and to 70% at 20–28 min. This composition was maintained until t = 35 min, before being reduced to the initial 0% B composition. The injection volume was 5 μL. The fluorescence intensity in the column eluate was monitored at 335 nm (excitation) and 439 nm (emission).

#### Metabolic stability

0.5 μL of **3** (2 mM in DMSO stock solution) was diluted with PBS (432 μL) and a 13 μL aliquot of Sprague − Dawley rat liver microsomes (Sigma-Aldrich, No. M9066) was added. The tube was vortexed at 37 °C for 5 min, then NADPH (50 μL, 10 mM in PBS stock solution) was added. The mixture was incubated at 37 °C for 60 min, and quenched by 250 μL of ice-cold CH_3_CN and centrifuged at 6000 rpm for 10 min. The supernatant was then analyzed by PDA-HPLC on Atlantis dC18 column (250 × 4.6 mm id, 5 μm particle size) (Waters). The HPLC column was eluted at a flow of 1 mL/min using a mixture of CH_3_CN and Milli-Q H_2_O (70:30). The injection volume was 5 μL. The procedure was repeated at least three times, and verapamil was used as positive control.

#### Cell cultures

The rat cell line CTX/TNA2 (normal astrocytes) was provided by the European Collection of Cell Cultures (ECACC, Merck, Darmstadt, Germany) and maintained in DMEM High glucose supplemented with 10% of FBS and 1% penicillin–streptomycin (100 μg/mL) (EuroCloneSpA Life-Sciences-Division, Milano, Italy) according to the ECACC’s instructions. Cells grew at 37 °C in a humified atmosphere of 5% CO_2_.

C6 rat glioma cells (ECACC®, Merck, Darmstadt, Germany) were cultured in HAM’s F-12 supplemented with 1% L-Glutammine, 10% FBS and 1% penicillin/streptomycin (EuroClone S.p.a., Milan, Italy). Cells were maintained at 37 °C and 5% of CO_2_ and sub-cultured as recommended by ECACC®.

#### Assessment of cell viability (MTT assay)

Cell viability was assessed by the MTT (3-(4,5-Dimethylthiazol-2-yl)-2,5-Diphenyltetrazolium Bromide) test (Merck, Darmstadt, Germany). Cells were seeded (8 × 10^3^/well) in a 96-well tissue culture-treated plate (Falcon®, Corning Incorporated, NY, USA) and let them adhere for 24 h. In the first set of experiments, both CTX and C6 cells were exposed to increasing concentrations of compound **3** (0–250 µM) up to 72 h. In a second set of experiments, C6 rat glioma cells were exposed to TMZ alone at 500 µM or to compound **3** (0–250 µM) and TMZ at 500 µM in combination. After exposure time, the MTT assay was performed as previously reported [25].

#### Cytotoxicity assay (LDH release)

The release of LDH into cell supernatants was quantified by the CytoTox 96® nonradioactive assay (Promega Corporation, Fitchburg, WI, USA) to assess cytotoxicity in both CTX and C6 cells after a 24 h exposure period. Cell supernatants analyzed were collected from cultures analyzed by the MTT assay. The assay was carried out as previously reported [15].

## Supplementary Information

Below is the link to the electronic supplementary material.

Supplementary file1 (DOCX 261 KB)

## Data Availability

Data will be made available on request. The authors declare that the data supporting the findings of this study are available within the paper and its Supplementary Information files. Should any raw data files be needed in another format they are available from the corresponding author upon reasonable request. Correspondence and requests for materials should be addressed to C.M.
