# Supplementary material for: Discovery of N-[2-(4-methylquinolin-2-yl)phenyl]acetamidine as a new potent nitric oxide synthase inhibitor against glioma progression
Source: Mol Divers. 2025 Aug 5;30(5):6977–84. doi: 10.1007/s11030-025-11309-0 (PMC13635051; doi:10.1007/s11030-025-11309-0)
Supplement: Supplementary file 1 [file 11030_2025_11309_MOESM1_ESM.docx]

**Discovery of N-[2-(4-methylquinolin-2-yl)phenyl]acetamidine as a new potent nitric oxide synthase inhibitor against glioma progression**

*Marialucia Gallorini, Rosa Amoroso, Amelia Cataldi, Cristina Maccallini**

*Department of Pharmacy, University “G. d’Annunzio” of Chieti-Pescara*

*corresponding author: cristina.maccallini@unich.it

CONTENT

Table S1

NMR spectra of compound 3

Table S2. Compound 3 reaction yields in different experimental conditions.

| Entry | Solvent | Temperature | Yield % |
| --- | --- | --- | --- |
| 1 | EtOH | r.t. | - |
| 2 | EtOH | 50° C | < 10 |
| 3 | EtOH | b.t. | 18 |
| 4 | free | r.t. | 13 |
| 5 | free | 50° C | 24 |
| 6 | free | 90 °C | 56 |

Compound 3 NMR spectra
